# Preliminary Indoor Radon Measurements Near Faults Crossing Urban Areas of Mt. Etna Volcano (Italy)

**DOI:** 10.3389/fpubh.2019.00105

**Published:** 2019-05-03

**Authors:** Marco Neri, Salvatore Giammanco, Anna Leonardi

**Affiliations:** ^1^Istituto Nazionale di Geofisica e Vulcanologia, Osservatorio Etneo, Catania, Italy; ^2^Freelance Geologist, Aci Castello, Italy

**Keywords:** radon, Etna, indoor pollution, human health, cancer

## Abstract

The slopes of Etna are crossed by numerous active faults that traverse various towns and villages. These faults pose a two-fold problem for the local people: on one hand, they cause frequent damage to houses and breakage of roads, while on the other they constitute a preferential route for the rising of crustal and sub-crustal gases, including radon, toward the surface. Various recent studies on the volcano confirm a high level of radon degassing measured both in the soil (> 10,000 Bq/m^3^), and inside homes (> 2,000 Bq/m^3^). For this reason, we felt the need to deepen our knowledge on the radon present in the Etnean area, focusing in particular on indoor radon pollution that, as widely recognized, is among the main causes of cancer largely (but not exclusively) of the respiratory system. Firstly, since 2005 we made a broad surface survey that revealed very high radon emissions from soils near active faults on Etna. Typical background soil activity on Etna were <1,000 Bq/m^3^, whereas in areas of stronger soil degassing, activity values up to ~60,000 Bq/m^3^ were measured. Furthermore, since late 2015 we have performed continuous indoor radon monitoring inside seven houses, some of which located close to degassing faults on the eastern, southern and south-western flanks of the volcano. Indoor radon concentration varied according to the season of the year, but above all, they changed according to the geology and tectonic setting of the substratum of the monitored houses. In one case, indoor radon concentration reached 3,549 Bq/m^3^ and remained > 1,000 Bq/m^3^ for several consecutive months, highlighting a potential health problem for those living in such environments. In other cases, the construction features of the houses and/or the materials used seemed to play an important role in the mitigation of indoor radon accumulation, even in the presence of intensely degassing soils. These preliminary data demonstrate the need to deepen the studies, extending indoor radon measurements to other urban areas, in order to monitor the health hazard for the Etna population, amounting to about one million people.

## Introduction

Radon (^222^Rn) is a short-lived decay product of uranium (^238^U). It is a noble gas, invisible, odorless, tasteless, and chemically inert. Radon is widespread in the Earth's crust, with activity concentrations ranging many orders of magnitude, chiefly depending on the original uranium content, on the physical characteristics of the rocks and on the ways of its motion through the rocks and soils. It is eight times heavier than air and this fact, together with its short half-life (3.8 days), limits its diffusivity to nearly 2 m in soil and 2 mm in water (1, 2). Therefore, high activity concentration in radon emissions at the topographic surface is produced by convective flow of gases that facilitate the transport of radon from greater depth within soils.

Faults and fractures of the Earth's crust are the easiest paths for radon to move through the rocks and get to the surface because these zones are generally characterized by a higher porosity than surrounding rocks. Indeed, faults are likely the locations of high soil degassing and elevated radon activities (3–7).

**Figure 1:**
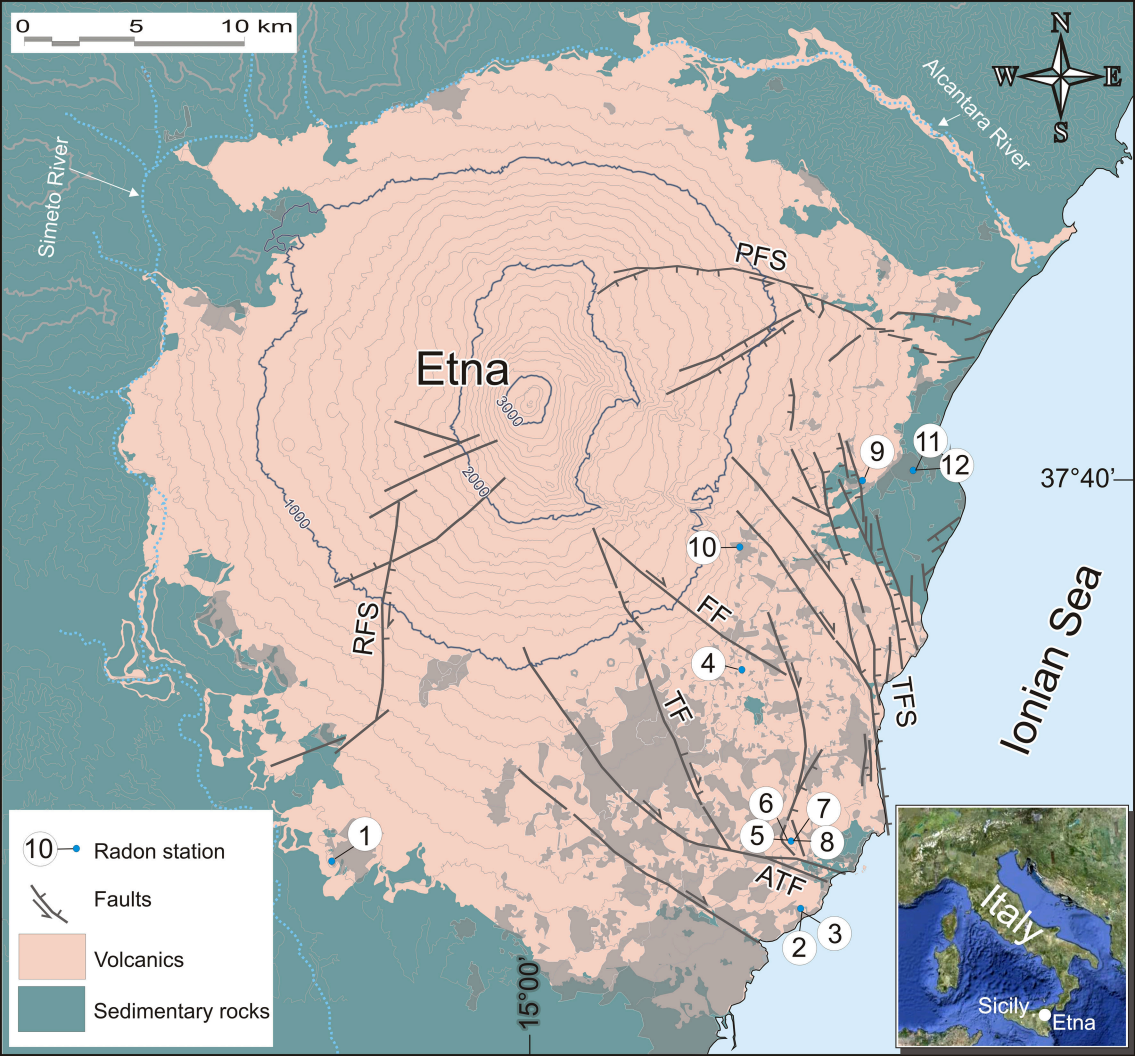
Simplified geological map of Mount Etna showing the main faults that cross the volcano and the sites of indoor radon measurements, numbered from 1 to 12. The urbanized areas are shown in light gray. Contour lines are in meters. PFS, Pernicana fault system; RFS, Ragalna fault system; TFS, Timpe fault system; FF, Fiandaca fault; TF, Trecastagni fault; ATF, Aci Trezza fault.

In recent years, many in-soil radon measurements were undertaken on Mt. Etna volcano (Italy), one of the most active volcanoes in the world (8) (Figure 1). The typical background level of in soil radon activity concentration on Etna was found to be <1,000 Bq/m^3^ (9–11), whereas in areas of stronger soil degassing radon activity was up to 60,000 Bq/m^3^ (12). Anomalous soil degassing is particularly strong in the east and southwest flanks of the volcano, that are characterized by continuous tectonic deformations and gravitational collapses (9, 13–15). The collapsing flanks are bordered by numerous active faults (10, 16–19), sometimes revealed from radon in-soil investigations (11, 12, 20–22), and many of these faults cross urban areas (Figure 1).

However, while proving very useful for monitoring faults and volcanic activity, radon emissions can be severely detrimental to human health. Continuous exposure to high concentrations of radon in poorly ventilated areas such as homes and workplaces poses radiation risk for those people inhaling radon gas. The short-lived radon progeny are alpha emitters and decay, most possibly inside human lungs before being exhaled. From the short-lived radon progeny, are alpha emitters with short half-lives of only 3 min and 164 μs, respectively, and therefore, they decay, most possibly inside human lungs before being exhaled. The radiation burden due the radon short-lived progeny has been associated to lung cancer incidence (23–27). Investigators (28, 29) have reported potential linkage to lymphocytic leukemia after long exposures.

Given the potential risk due to radon exposure, the strong radon emissions from the soils of the Etna area measured thanks to the recent investigations have led the local scientific community to start undertaking studies on the accumulation of this gas inside houses and public buildings in urban areas built in the nearby area. However, though limits on indoor radon exposure have been already set in various countries (30), in many densely populated volcanic areas, and particularly on Etna, systematic measurements of indoor radon concentrations had never been carried out so far and therefore the risk posed by radon was practically unknown. Various recent studies on Etna confirmed high levels of radon concentrations measured, for short periods, inside homes [> 2,000 Bq/m^3^, (31, 32)]. These values greatly exceed the annual average concentration reference level of 100 Bq/m^3^ suggested by the World Health Organization (33). WHO also declared that if this level cannot be reached under the prevailing country-specific conditions, the reference level should not exceed 300 Bq/m^3^, which is the limit recommended also in Europe by the EU Council Directive 2013/59/EURATOM.

For the above reasons, in this paper we present the first ever long-term monitoring of indoor radon on Mt. Etna. Measurements were carried out between 2015 and 2018 inside seven houses built on different types of soil and at varying distances from faults. The selected houses were located on the eastern, southern and south-western flanks of Etna, respectively (Figure 1). We compared the acquired data on radon activity with the geological settings at each site, in order to identify all possible sources of radon. In some cases, we found levels of radon activity potentially dangerous for the local inhabitants.

## Methods

We used a digital radon-monitor (model *Canary*, manufactured by Corentium AS, Oslo, Norway), that uses Alpha spectrometry with an Rn-accumulative method. The monitor calculates and records both the daily and the weekly average radon activity and it provides the cumulative radon activity concentration in the long term, up to 1 year. The instrumental activity range is from 0 to 9999 Bq/m^3^, with absolute accuracy <5%. Sensitivity at activity concentration of 100 Bq/m3 is ~1.3 pulses/hour for 1-day-short-term measurement and ~0.3 pulses/hour for 7-days-long-term measurement. The radon-monitors were placed in rooms connected to underground spaces, 50 cm above the floor and at least 150 cm away from the next aeration facility.

We selected seven locations at various altitudes (from 42 to 592 m above sea level) on the eastern and southern slopes of Mt. Etna, chosen in places where high radon activity concentration had been measured in soils during previous surveys (10, 12, 13, 34). In three of those locations, we used several active radon monitors together during the period of our investigation, to evaluate possible concurrent temporal variations of radon activity in different rooms. Homes and school buildings were selected also based on the inhabitants' willingness to collaborate voluntarily with data collection.

Radon measurements were performed in a fully automated way. However, changes in radon activity had to be transcribed to a spreadsheet by the operator day by day, since the instruments permanently stored only the average value of radon activity (in the long–term mode). Two schools were also involved in our surveys. The school-age young persons, before being involved as operators, were preliminarily instructed with seminars on radon gas and its potential effects, both positive (as tracer of geodynamic activities) and negative (as a risk factor for lung cancer).

Twelve *Canary* digital radon-monitors were used at the seven locations above, with measurements taken during different intervals from 11/12/2015 until 20/10/2018. In some of the seven locations, more instruments were placed in order to monitor different rooms (Table 1). All rooms were without a basement underneath.

**Table 1:** Summary of the radon measurements carried out between the end of 2015 and October 2018 in homes and schools located on the slopes of Etna.

													^**222**^**Rn-1 Day**		^**222**^**Rn - Long term**			
**Location #**	**Location name**	**Sensor #**	**Lat**	**Lon**	**Altitude m a.s.l**.	**Floor**	**Type of room**	**Lithology of basement rocks tickness in m**	**Distance from fault m**	**Days of meas**.	**Starting time**	**Ending time**	**Min Bq/m^**3**^**	**Max(Bq/m^**3**^)**	**Min Bq/m^**3**^**	**Max Bq/m^**3**^**	**Average Bq/m^**3**^**	**Annual dose mSv/y**
1	Paternò	1	37.57	14.89	217	Ground	Living room	Thin lavas and scorias (1–5 m) on sands and silty-clays (>100 m)	5000	783	20/02/16	12/04/18	0	87	12	58	35	0.83
2a	Aci Castello, Cannizzaro	2	37.54	15.13	43	Ground	Bedroom	Lavas and scorias (60–100 m) on silty-clays (>200 m)	1800	869	18/03/16	03/08/18	5	605	74	346	**110**	1.45
2b	Aci Castello, Cannizzaro	3	37.54	15.13	43	Mezzanine	Bedroom	Lavas and scorias (60–100 m) on silty-clays (>200 m)	1800	1044	11/12/15	20/10/18	1	669	37	360	91	1.20
3	Zafferana Etnea, Pisano	4	37.67	15.11	446	Ground	Living room	Lavas and scorias (>400 m)	1200	595	28/02/17	17/10/18	12	330	125	187	**159**	3.76
4a	Aci Catena	5	37.57	15.14	215	Basement	Bedroom	Bavas and scorias (>200 m)	150	625	01/01/16	16/09/17	3	988	165	988	**230**	5.44
4b	Aci Catena	6	37.57	15.14	215	Basement	Storage room	Lavas and scorias (>200 m)	150	582	13/02/16	16/09/17	5	623	87	357	**192**	1.26
4c	Aci Catena	7	37.57	15.14	215	Basement	Kitchen	Lavas and scorias (>200 m)	150	43	01/01/16	12/02/16	118	452	200	325	**315**	7.45
4d	Aci Catena	8	37.57	15.14	215	Basement	Living room	Lavas and scorias (>200 m)	150	43	01/01/16	12/02/16	146	243	111	329	**231**	5.46
5	Macchia di Giarre	9	37.72	15.17	189	Ground	Bedroom	Lavas and scorias (>100 m)	40	890	27/04/16	03/10/18	0	3549	170	1318	**524**	6.89
6	Zafferana Etnea	10	37.69	15.11	592	Ground	Classroom	Lavas and scorias (>600 m)	1600	37	03/05/18	08/06/18	12	131	31	131	31	0.24
7a	Giarre, classroom1	11	37.73	15.19	56	Ground	Classroom	Pebbles and gravels in sandy to silty matrix (>50 m)	2300	105	18/02/17	02/06/17	4	49	30	89	30	0.24
7b	Giarre, classroom 2	12	37.73	15.19	56	Ground	Classroom	Pebbles and gravels in sandy to silty matrix (>50 m)	2300	134	18/01/18	31/05/18	6	102	33	68	54	0.43
Averages					209					479			26	652	90	380	167	2.89

*The lithological characteristics of the substrate, the distance from the nearest known faults and the average annual radon doses are also indicated. The columns on the right indicate the daily radon averages and those for long–term monitoring. Long-term radon activity concentration higher than 100 Bq/m^3^ are highlighted in bold*.

## Results

### Municipality of Paternò

The radon-monitor was placed in a house at the SW periphery of the Paternò town, close to an area characterized by the presence of mud volcanoes (35, 36). The origin of those mud volcanoes is probably related to a structural trap formed by a tectonic fold of Pleistocene clays inside the sedimentary basement of Etna. Fluids ascend through pre-existing volcanic necks and/or through a NE-SW regional fault. CO_2_ is the most abundant escaping gas, and it is predominantly of deep magmatic origin (35, 37–40).

**Figure 2:**
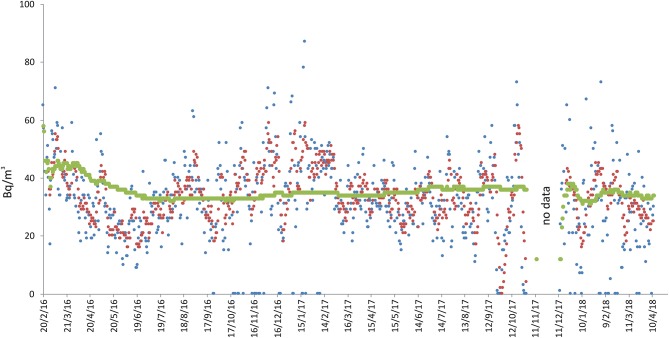
Radon measurements recorded at site 1 in Paternò (see Figure 1 for location). Radon concentration are presented as daily (blue line), weekly (red) and long-term (green) averages. “No data” indicates periods of no acquisition due to battery failure.

Local geology under the house consists of thin lavas and scoriae (1–5 m) > 15 kyr in age, covering sands and silty-clays (at depth > 100 m). The nearest known fault is at a distance of about 5 km (RFS in Figure 1). The load-bearing structure of the house is in reinforced concrete, with bricks in the infill walls. The radon-monitor was installed in a corner of the living room on the ground floor, about 50 cm above the floor. Measurements started on 20 February 2016 and are still ongoing, even if here we present data until April 2018 (Figure 2). At this site, radon fluctuations ranged from 0 to 87 Bq/m^3^, with long-term average concentration of 35 Bq/m^3^, and it is worth noting that during winter periods radon oscillations were greater.

### Municipality of Aci Castello

Two radon-monitors were placed inside a house located at the southern boundary of the municipality of Aci Castello, at an elevation of 43 m above sea level, respectively on the ground floor and mezzanine. The house is standing on > 60 m of compact lava flows and scoriae erupted during the last 10 kyr. The nearest known fault is located at a distance of about 1.8 km (Aci Trezza fault in Figure 1). The house was built with load-bearing walls made up of blocks of lava and cement mortar. In both cases, the radon-monitors were installed inside bedrooms, about 50 cm above the floor.

**Figure 3:**
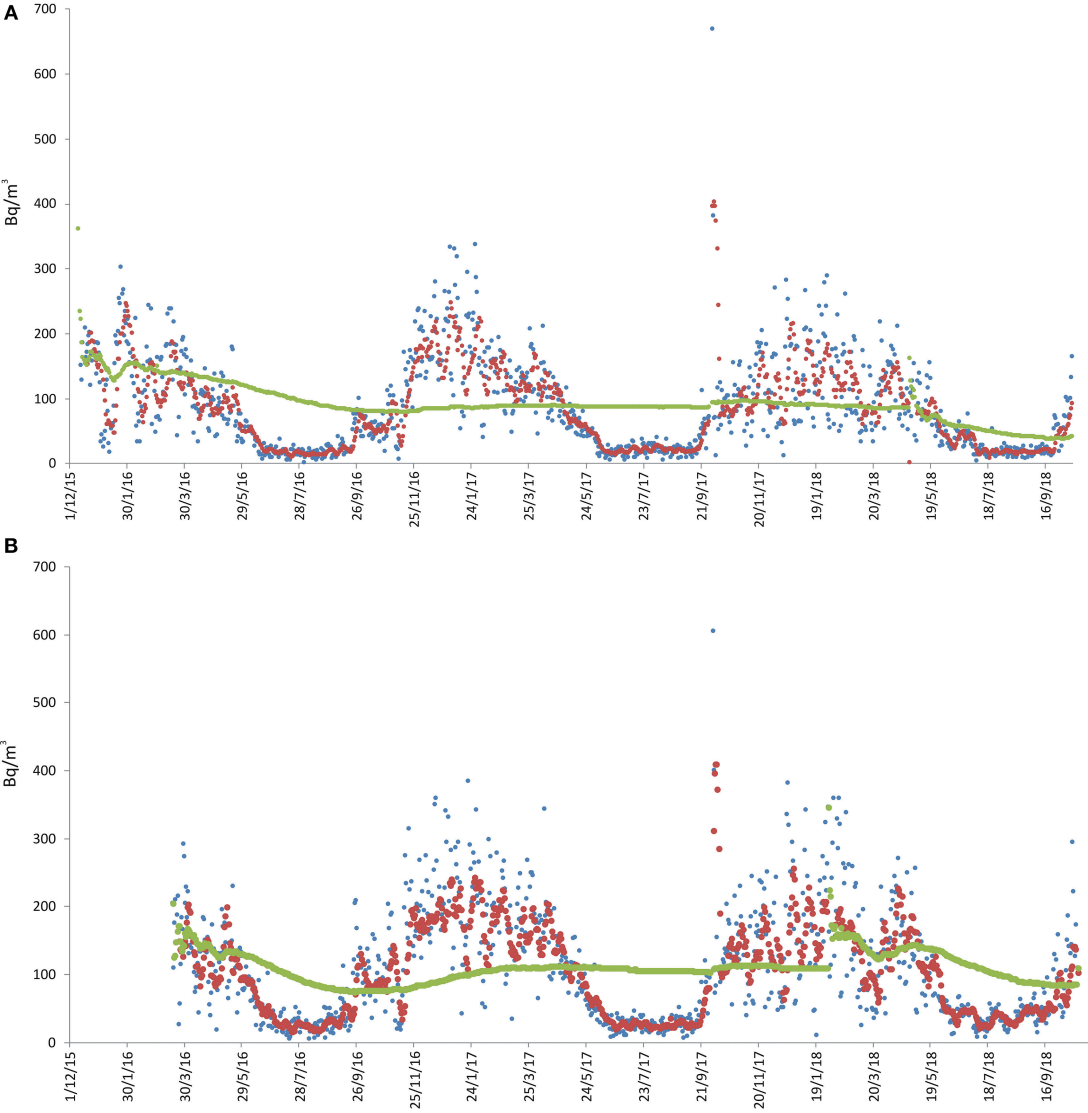
Radon measurements recorded at location 2 in Aci Castello [see Figure 1; **(A)** = sensors 2, **(B)** = sensor 3]. Radon concentration are presented as daily (blue line), weekly (red) and long-term (green) averages. The remarkable peaks of radon activity were recorded on 4 October 2017, after 3 days when the house remained closed.

The measurements started on 11 December 2015 and are still ongoing (Figure 3). In these two sites radon activity concentration ranged from 5 to 605 Bq/m^3^ (ground) and 1 to 669 Bq/m^3^ (mezzanine), with long-term averages of 91 and 110 Bq/m^3^, respectively, the higher long-term concentration being recorded by the monitor on the ground floor, as expected. The highest radon value (669 Bq/m^3^) was recorded on 4 October 2017 at the mezzanine, after keeping the house completely closed for 3 days. Radon concentration consistently higher than 300 Bq/m^3^ were recorded only for about 2 consecutive months, during the winter season. The two instruments showed substantially synchronous fluctuations, with definitely higher concentration from autumn to spring (from October to May), while during the summer the radon dropped to almost zero. Every time the instrument batteries were changed, the sensors were reset and for this reason, in the days immediately following, higher concentration were recorded compared to the previous period. This instrumental behavior is consistent with the *Canary* radon-monitor instructions, as the instrument needs about a week to stabilize.

### Municipality of Zafferana Etnea

Two radon-monitors were placed in two different houses. Site 4 is located at the southern periphery of the municipality of Zafferana Etnea, in a zone named “Pisano,” at 446 m above sea level, in a living room on the ground floor. Under the house there is a very thick (presumably > 400 m) succession of lava and scoria layers. The building is standing on scoriae, about 2 meters thick. The nearest known fault is located at a distance of about 1.2 km (Fiandaca fault in Figure 1). The house is built of reinforced concrete.

**Figure 4:**
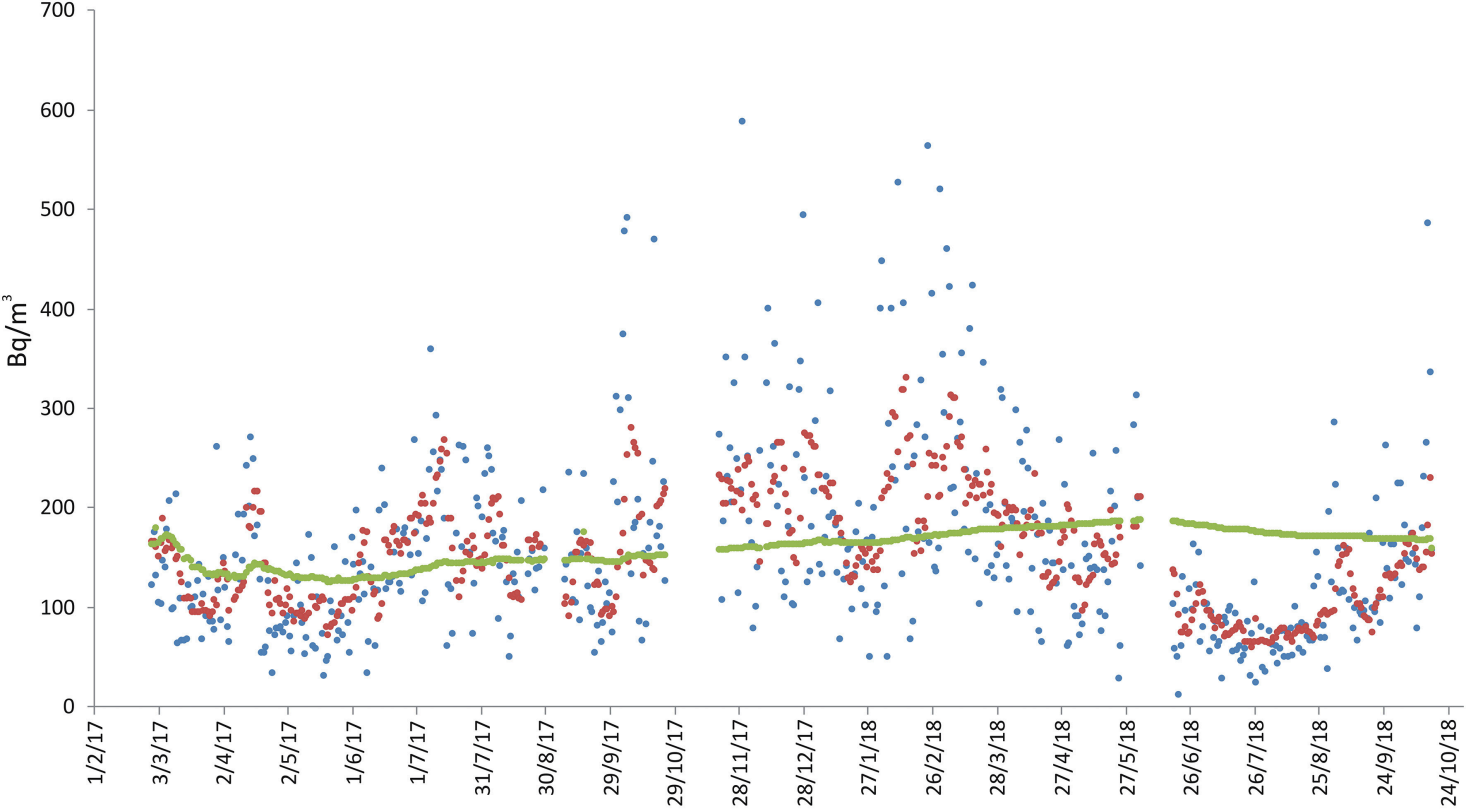
Radon measurements recorded at location 3 (see Figure 1, sensor 4), in the Zafferana Etnea territory. Radon concentration are presented as daily (blue line), weekly (red), and long-term (green) averages. No data interruption occurred during the acquisition period. The short discontinuities of the green line (long-term average) indicate only discontinuity in the data annotation.

At this site, measurements started on 28 February 2017 and are still ongoing (Figure 4). Radon ranged from 12 to 587 Bq/m^3^, with a long-term average value of 159 Bq/m^3^. A slight cyclicity linked to the alternation of the seasons is visible, with higher radon concentration (from about 300 to 600 Bq/m^3^) between October and April and lower concentration (<100 Bq/m^3^) between June and August 2018. However, this trend was apparently not followed during the summer of 2017, when the average daily radon activity was higher than expected. Activity concentration actually ranged between 100 and 200 Bq/m^3^, with a maximum of 358 Bq/m^3^ on 7 July 2017.

**Figure 5:**
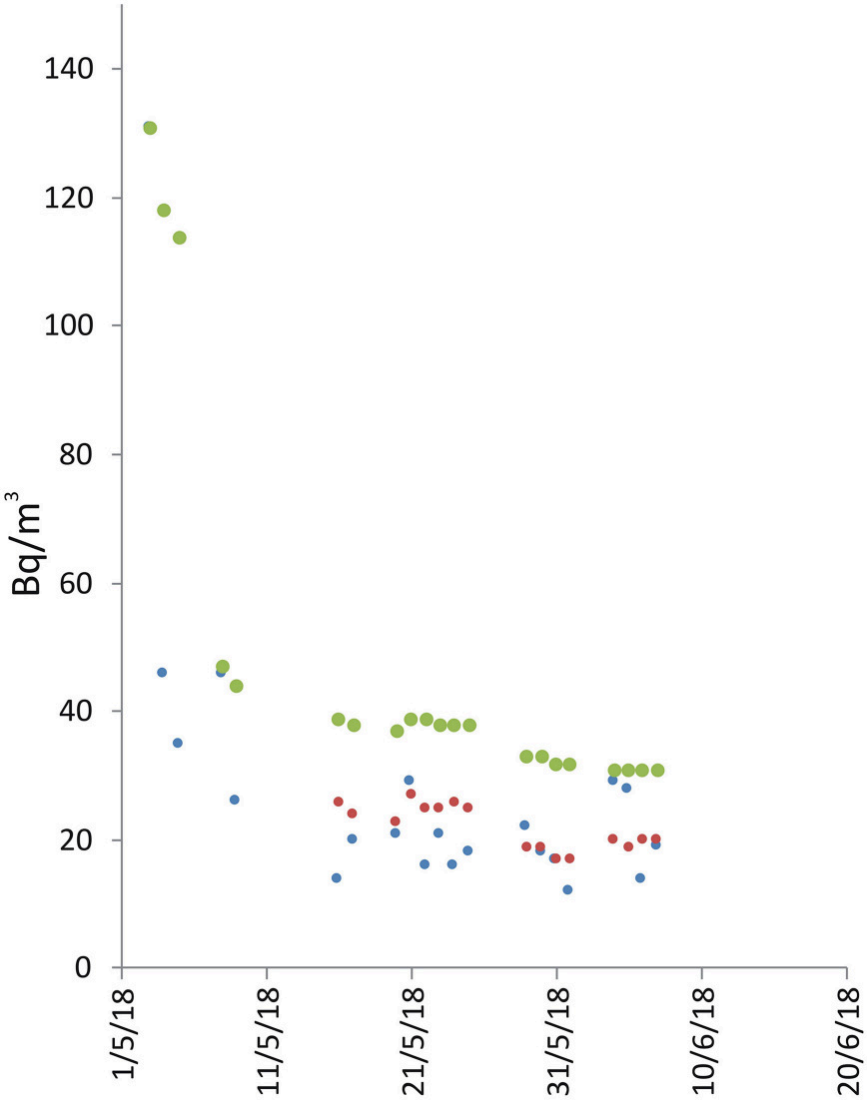
Radon measurements recorded in the Zafferana Etnea territory, at location 6 (see Figure 1, sensor 10). Radon concentration are presented as daily (blue line), weekly (red), and long-term (green) averages. The discontinuities of the green line (long-term average) indicate only discontinuity in the data annotation.

Site 10 is located in the center of Zafferana Etnea (592 m above sea level), in a classroom on the ground floor of a primary school. The edifice was built of reinforced concrete and it stands on a succession of lava flows over 600 m thick. The nearest known fault is located at a distance of about 1.6 km NE of the site (Figure 1). In this case we performed only about 1 month of radon measurements (from 3 May to 8 June 2018, see Table 1 and Figure 5), but a new radon survey was begun in mid-October 2018 and is still ongoing. The few measurements carried out showed low radon activity (from 12 to 131 Bq/m^3^, with a long-term average value of 31 Bq/m^3^).

### Municipality of Aci Catena

**Figure 6:**
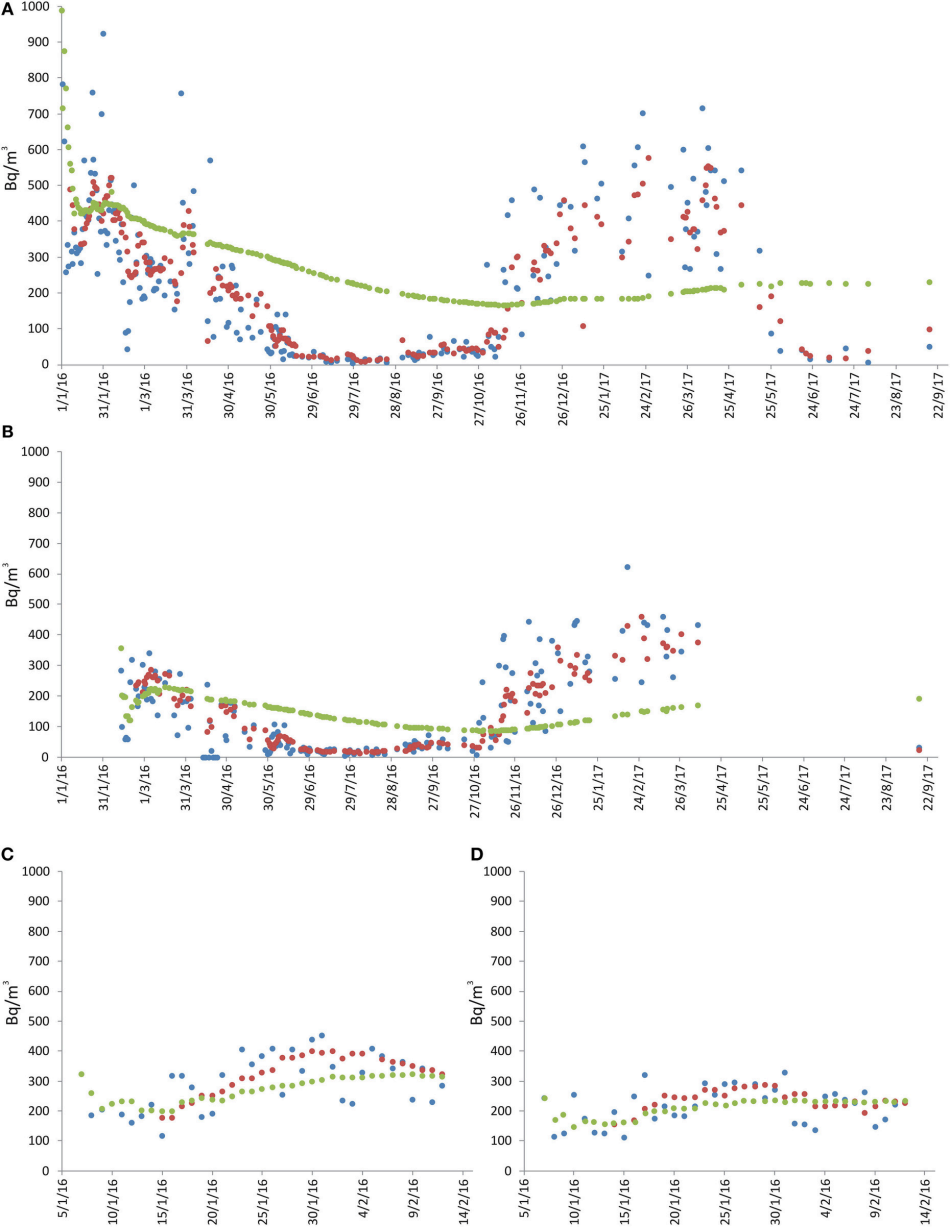
Radon measurements recorded in the Aci Catena municipality, at location 4 [see Figure 1; **(A)** = sensor 5, **(B)** = sensor 6, **(C)** = sensor 7, **(D)** = sensor 8]. Radon concentration are presented as daily (blue line), weekly (red) and long-term (green) averages. No data interruption occurred during the acquisition period. The discontinuities of the green line (long-term average) indicate only discontinuity in the data annotation. See text for details.

Four radon-monitors were placed in a house at the southern periphery of the municipality of Aci Catena (215 m above sea level), on the ground floor. The ground under the house is presumably made of > 200 m of lava flows and scoriae. The building is standing on a thin (about 1.5 m) lava layer, interbedded with volcanic scoriae. The house was built of reinforced concrete and bricks and the building is located very close to several active faults (~150 m from the closest one; see Figure 1). Measurements spanned from 1 January 2016 to 16 September 2017. We initially monitored four different rooms to get an overall picture of the situation, as the first radon measurements immediately showed high values. Once the substantial synchronicity of the measurements at all four sites had been established, we continued monitoring at only two sites (sites 5 and 6, bedroom and storage room, respectively. See Figure 6).

At these two sites, 1-day average for radon activity ranged from 3 to 988 Bq/m^3^ (bedroom) and from 5 to 623 Bq/m^3^, with long-term average concentration of 230 and 192 Bq/m^3^, respectively. Radon concentration consistently higher than 300 Bq/m^3^ were recorded from January to April 2016 and from October 2016 to May 2017 (Figure 6). The two instruments also showed synchronous fluctuations, but with higher radon concentration at site 5 (bedroom). Generally, higher values were recorded from autumn to spring, while during the summer radon activity dropped to almost zero, similar to what was observed at sites 2 and 3 in the Aci Castello municipality.

### Municipality of Giarre

Three radon-monitors were placed in two different localities: site 9 was inside a private house located in the northern suburbs of the town of Giarre, while sites 10 and 11 were in two different classrooms of a high school within the Giarre urban area.

**Figure 7:**
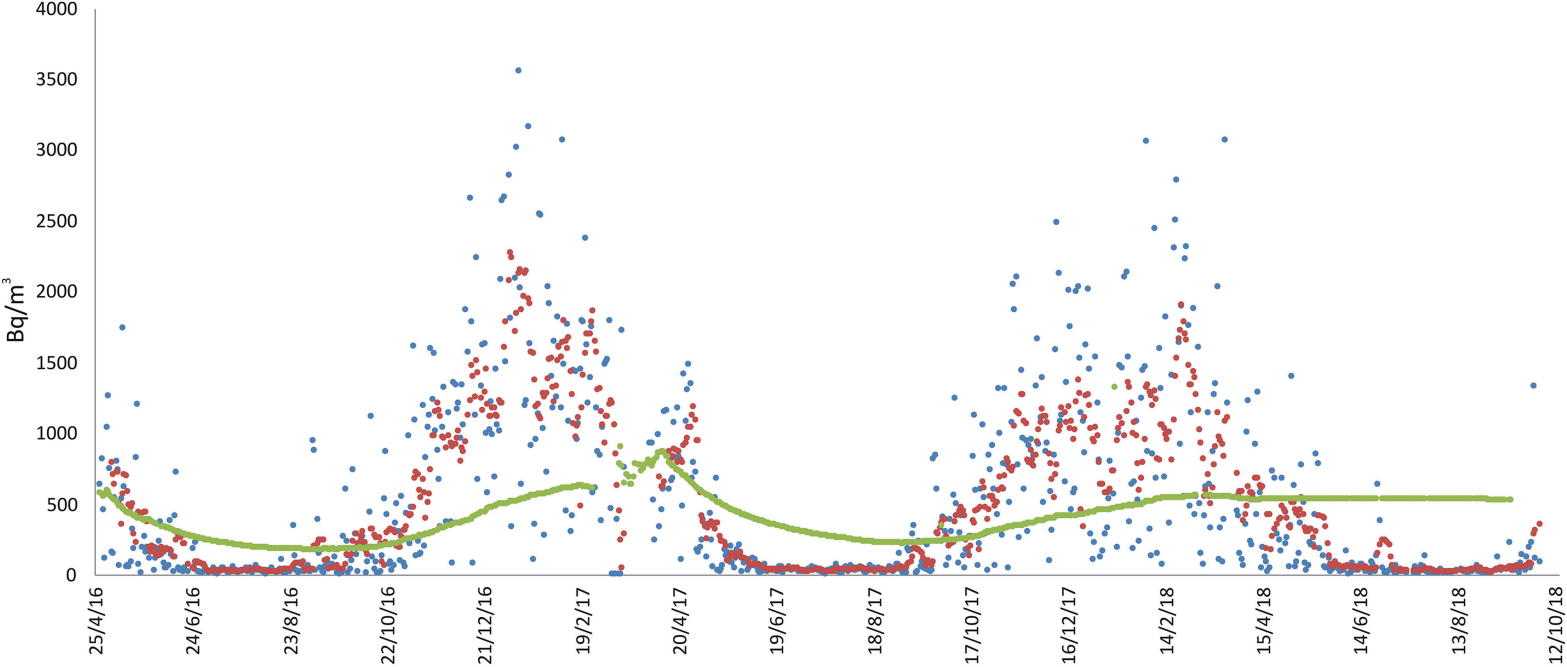
Radon measurements recorded in the Giarre municipality, at location 5 (see Figure 1, sensor 9). Radon concentration are presented as daily (blue line), weekly (red), and long-term (green) averages. No data interruption occurred during the acquisition period.

Site 9 was located at 189 m above sea level, the radon monitor being positioned in the bedroom on the ground floor. Under the house there are presumably > 100 m of lava flows and scoriae. The building is standing a very compact lava layer, crossed by fractures derived from contraction (during the cooling of the lava). The house is built of reinforced concrete and bricks, and is very close to an active fault (~40 m west of site; see Figure 1). The measurements started on 27 April 2016 and are still ongoing (Figure 7). Radon activity ranged from 0 to 3549 Bq/m^3^, with long-term average value of 524 Bq/m^3^. As for the previous sites, also in this case higher radon activity concentration (> 500 Bq/m^3^) were recorded for several months from autumn to spring, while during summer the radon concentration dropped to almost zero.

Sites 11 and 12 are located inside a school at 56 m above sea level. The radon monitors were positioned on the ground floor. The geological substratum is made of pebbles and gravels in a sandy to silty matrix, belonging to a stratigraphic formation known as “Chiancone,” an alluvial debris deposit formed approximately between 10,000 and 5,000 years ago (41). The building is standing directly on this pebbles and graves. The school was built of reinforced concrete and bricks, at a distance of 2,300 m from known active faults (Figure 1). The radon measurements were performed, respectively, between 18 January and 2 June 2017 at site 11 (classroom 1) and between 18 January and 31 May 2018 at site 12 (classroom 2; Figure 7). During these two periods, 1-day radon activity ranged from 4 to 49 Bq/m^3^ (classroom 1) and from 6 to 102 Bq/m^3^ (classroom 2), with long-term average radon concentration of 30 Bq/m^3^ (classroom 1) and 54 Bq/m^3^ (classroom 2).

## Discussion

Data were acquired with variable time spans, ranging from a few months to about 3 years, starting from early December 2015. This is the first ever attempt to perform a systematic and long-term (from a few months to over 3 years, see Table 1) near-continuous monitoring of indoor radon activity in the places where radon emissions had previously been measured with different instruments and in different sampling frequencies (10, 12, 13, 34). Continuous soil gas radon monitoring is being carried out on Mt. Etna since 2005 by the monitoring networks of the Istituto Nazionale di Geofisica e Vulcanologia, Osservatorio Etneo (INGV-OE). However, the INGV network measures radon activity in the subsoil (at 1.5–3 m depth), not at the surface, and outside urban areas because the main goal of those measurements is to use radon as a proxy to both tectonic and volcanic activity (11, 20–22, 42). On a few occasions, radon measurements in the soil along faults have also been performed in or near urban areas, but in a discontinuous way (9, 10, 12, 13). Only one study was recently devoted to detect the outdoor radon concentration in the Etna area (43), finding low activity levels in all sites at low altitude (range of 3.0–19.6 Bq/m^3^), particularly near the Ionian Sea on the east side of the volcano. Slightly higher radon concentration levels (up to 93 Bq/m^3^) were only found near the summit craters of the volcano. Finally, some indoor radon measurements have occasionally been carried out in houses on the eastern flank of Etna, but for very short periods (31, 44). The main contribution of this study is that it provides radon concentrations in residential homes, with measurements carried out for sufficiently long periods to be used to evaluate the risk on health from long-term radon exposure.

Studies on the carcinogenicity of radon (30, 33, 45) suggest thresholds above which the probability of lung cancer incidence increases significantly. These thresholds correspond to 100 Bq/m^3^ (attention threshold) and to 300 Bq/m^3^ (maximum threshold recommended). However, even if there is strong evidence of an association between indoor radon concentration at home and lung cancer, there is no evidence of a specific threshold dose. Indeed, recent studies show that the likelihood of lung cancer from radon exposure is linked by a linear relationship, especially if exposure to radon is added to active smoking (33, 45, 46). In other words, the risk of lung cancer can be high in the case of smokers who live in environments where radon activity concentration is even below the thresholds indicated above.

**Figure 8:**
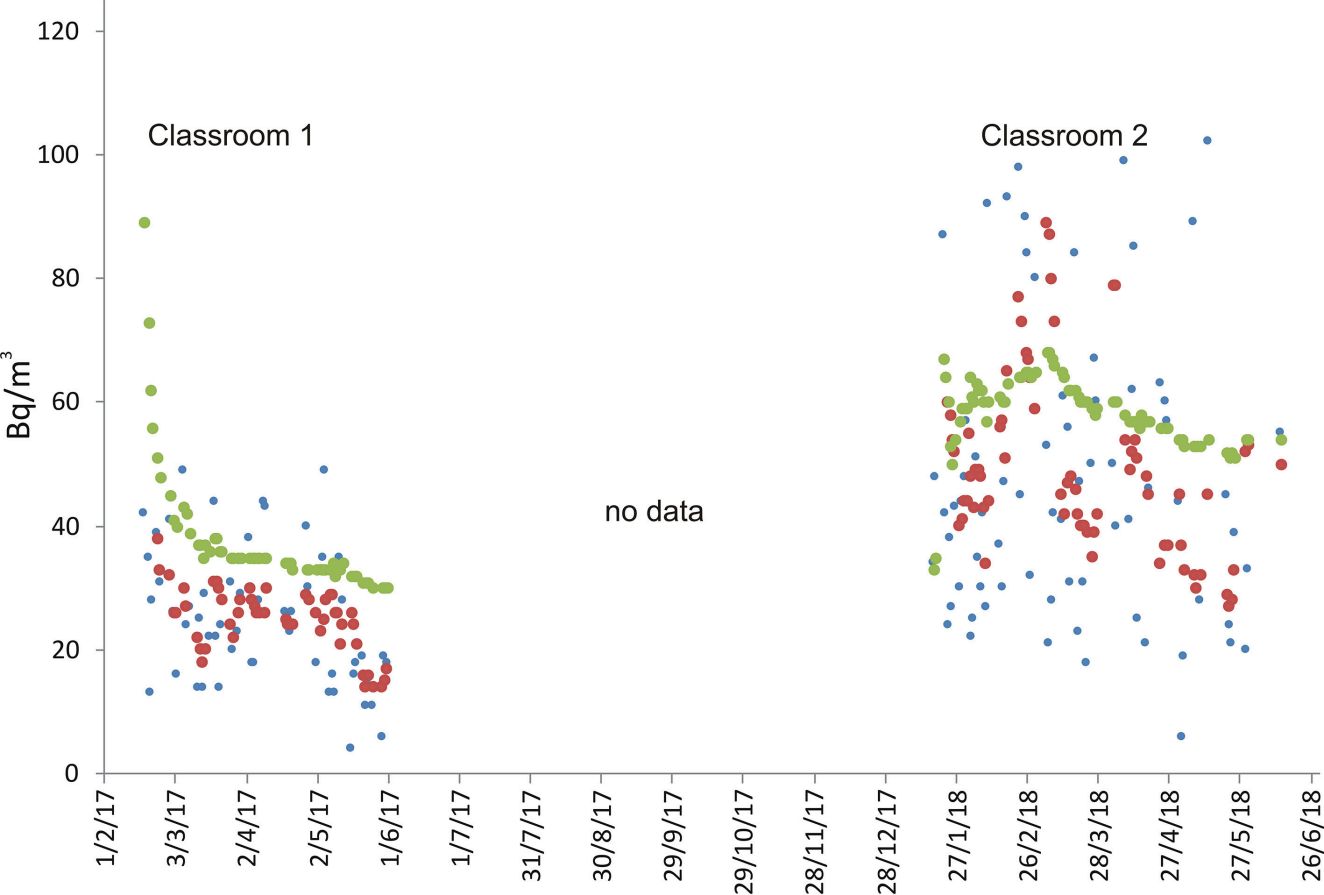
Radon measurements recorded in the Giarre municipality, at location 7 (see Figure 1, sensors 11 and 12) in two different classrooms inside a high school. Radon concentration are presented as daily (blue), weekly (red), and long-term (green) averages. “No data” indicates periods of no acquisition.

This study is a preliminary approach to estimate indoor radon concentrations in residential dwellings of the Etna region through long-term measurements. However, the possibility of recording daily and weekly mean radon concentration allows highlighting phenomena potentially induced by particular endogenous events, such as eruptions and seismic crises, since we are monitoring an active volcano. For this reason it is useful to represent daily and weekly concentration in the diagrams (see Figures 2–8), even if this part of the data is not widely discussed because it would be off topic in this paper.

Among the 12 radon monitors used in this study, seven showed mean long-term concentration > 100 Bq/m^3^, namely the first level of attention for annual average exposure recommended by the WHO handbook (33). In five cases, measurements were carried out for more than a year consecutively, and in one case for over 3 years (see Table 1). In these five cases, the monitored buildings are located in the municipalities of Aci Castello (site 3), Zafferana Etnea (site 4), Aci Catena (sites 5 and 6), and Giarre (site 9). Sites 7 and 9 showed long-term radon concentration > 300 Bq/m^3^, the maximum threshold recommended by the WHO handbook (33) and by the EU Council Directive 2013/59/EURATOM. In one case, indoor radon concentration were even > 1,000 Bq/m^3^ for long periods (months), and high radon accumulation (up to 669 Bq/m^3^ in sensors 2 and 3) was recorded during the period when the house was kept completely closed for a few days, which demonstrates the importance of a frequent ventilation of homes. Therefore, several of these case studies need to be evaluated with particular attention to safeguard the occupants' health. Further studies are being carried out to analyse the same radon data with the aim of identifying the causes that determine the highest indoor radon concentrations. In terms of doses, we estimated the annual mean effective dose H (in mSv/y) at each location using the procedure indicated by UNSCEAR (47, 48). We used the following equation:

(1) $$\begin{array}{r} \text{H}=\text{C}\times \text{E}\times \text{F}\times \text{T}\times \text{D} \end{array}$$

Where C is the average radon concentration measured at each location during the respective monitored period, E is an equilibrium factor (set at 0.4), F is the occupancy factor calculated as the percentage of occupation time of each location as a function of the type of room (varied between 0.20 and 0.75), T is the number of hours in a year and D is a dose conversion factor (set at 9.0 × 10^−6^ mSv and expressed in Bq/m^3^ h). The resulting values (Table 1) ranged from 0.24 to 7.45 mSv/y, with an average of 2.89 mSv/y. These figures are relatively high compared to other areas such as Japan (49), but they fall within the typical world range of doses due to inhalation of natural radiation sources for internal exposure [1-10, according to UNSCEAR (48)] and the average value corresponds well with the estimated worldwide annual effective dose per person [2.40 mSv, UNSCEAR (48)].

**Figure 9:**
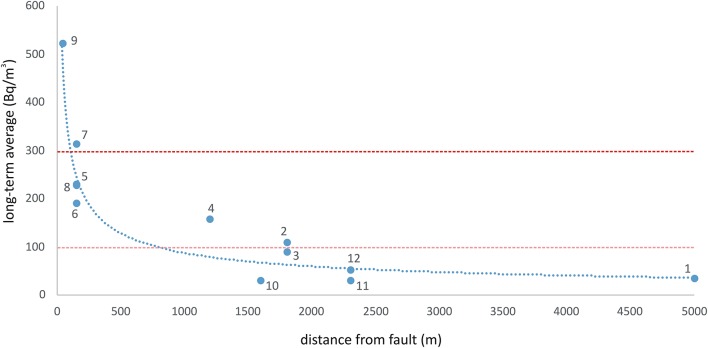
Correlation between long-term average radon and distance from known active faults. Sites 7 and 9 showed radon activities that exceeded the maximum threshold (300 Bq/m^3^) recommended by the WHO handbook (33) - represented by the red dashed line. The pink dotted line indicates the limit of 100 Bq/m^3^ recommended as the attention threshold by the WHO. The dotted blue line represents the best-fit model to the data (following a power law: y = 3,928 × ^−0.551^, with *R*^2^ = 0.8).

As a possible explanation for the dosimetric findings of this preliminary study, we have to consider that the houses showing the highest radon concentration are located at short distances (40–150 m) from active faults with visible surface cracks and fractures. In all other cases, the distance of the houses from known faults is > 1,000 m (Figure 9). This would suggest the local tectonics, and consequently the presence of active faults close to the monitored buildings, play a major role in the ascent of radon gas to the surface and its consequent infiltration into nearby homes. This might be a key factor for future monitoring of indoor radon in existing homes and for planning further urban expansion of cities.

As a further outcome of our study, we noted that the type of geological substrate under the monitored buildings had a clear influence on indoor radon activity, since the highest radon concentration were recorded in the sites located over successions of lava flows > 60 m thick (see Table 1).

**Figure 10:**
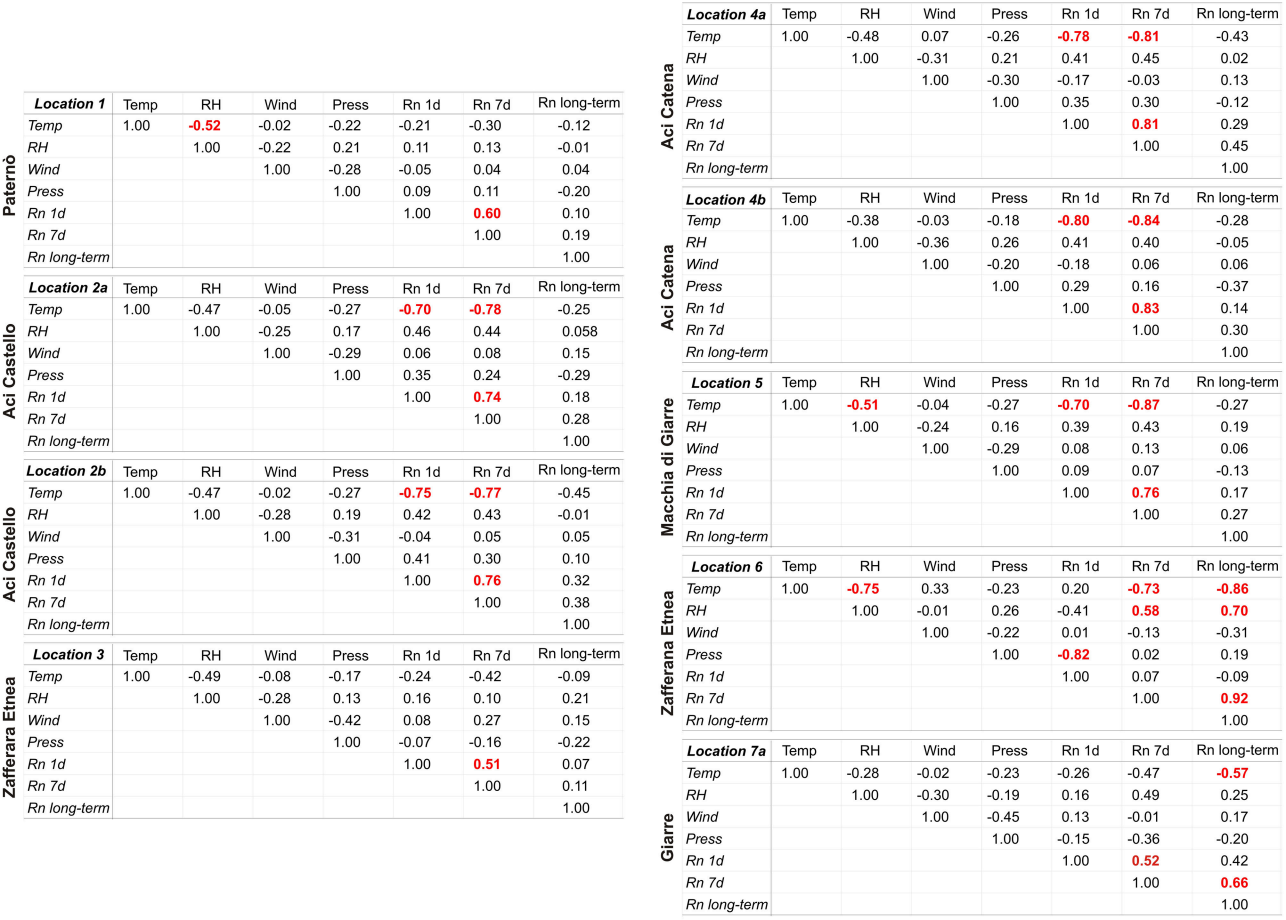
Correlation matrixes between average daily, weekly and long-term radon activity concentrations (indicated, respectively, with Rn 1d, Rn 7d, and Rn long-term) and the corresponding average daily values of air temperature (Temp), air relative humidity (RH), wind speed (Wind), and barometric pressure (Press) at the locations with the longest periods of indoor radon monitoring. Values of the Pearson correlation coefficient higher than ± 0.50 are considered statistically significant and therefore were highlighted in red.

Another factor that apparently heavily influenced indoor radon concentration was seasonality, especially at the sites monitored for the longest time (Figures 3, 6, 7). In those sites, a higher radon activity concentration was generally observed during periods with lower air temperature (fall and winter seasons). This finding is in good agreement with the results obtained in other parts of the world [e.g., Oikawa et al. (49); Groves-Kirkby et al. (50)]. In order to analyse statistically the possible influence of meteorological parameters on radon emission and diffusion into houses, we correlated the average daily, weekly and long-term radon concentrations with the corresponding daily averages of the meteorological parameters that seemingly most affect radon activity (i.e., air temperature, air relative humidity, wind speed, and barometric pressure). Meteorological data were measured at the Catania Airport and were provided by ilMeteo.it (www.ilmeteo.it). Figure 10 shows the correlation matrixes for all of the above parameters at each monitoring location where the radon time series was long enough to allow for a meaningful statistical correlation (i.e., 1, 2a, 2b, 3, 4a, 4b, 5, 6, 7a). The results show an obvious positive correlation between 1-day and 7-day radon averages at all sites except 6, where the best correlation was between 7-day and long-term radon averages. Another obvious correlation is found at almost all sites (though sometimes with Pearson coefficient values slightly lower than the minimum significance threshold of ± 0.50) between air temperature and relative humidity, with opposite sign because when air temperature is higher relative humidity decreases significantly. Furthermore, almost all sites show a strong negative correlation between 1-day or 7-day radon averages and daily average air temperature. Location 6 is the only one where we found a significant negative correlation between daily radon averages and daily average barometric pressure and significant positive correlations among 7-day, long-term radon averages and daily average relative humidity. Conversely, location 1 is the only one without any significant correlation among radon activity and any meteorological parameter.

Negative correlations between radon concentration and air temperature could be caused by changes in the air density associated with changes in air temperature. High temperature—and hence low density—of air promotes upward and outward convective motions of air in the houses, with consequent lowering of indoor radon concentration. The opposite occurs during low-temperature periods. However, the same effect could also be caused, at least in part, by the behavior of the inhabitants. People are actually induced to ventilate the rooms more frequently during periods of intense heat, typical of Sicilian summers, thus limiting the possibility of accumulation of radon inside rooms. On the other hand, no seasonal cyclicity due to meteorological parameters was clearly observed in the radon concentration measured at locations 1 and 4. In the case of site 1, a marginal cyclicity linked to seasonality probably appears when considering some wider oscillations of the daily summer values (Figure 2). However, it should also be noted that this site is located close to an area characterized by the presence of mud volcanoes, where changes induced by the remarkable flux of gas ascending from the subsoil (the gas pressure certainly being higher than atmospheric one, as it causes prominent bubbling in the mud pools) can prevail over the effects induced by variations in atmospheric pressure. Site 4, instead, probably showed variations of indoor radon that were induced by the tectonic and/or volcanic activity that characterized Etna in the spring of 2017 onwards (51). Absence of correlation between radon concentration and meteorological parameters at location 7 could be explained in a similar fashion as for location 1, because this site is very close to an active fault (Figure 9). Lastly, at location 5 (sensor 9) the influence of air temperature on radon concentration, though strong, was apparently not enough to prevent radon from accumulating inside of the house even during the warmest periods, due to the very short distance from an active degassing fault (Figure 9).

The construction typology of the monitored buildings is mainly reinforced concrete and brick pillars.

In one case only (sensors 2 and 3), the selected house was made of load-bearing walls and lava stone. Although this house is located far from known active faults (the nearest is about 1,800 m away), radon activities were quite high (up to over 300 Bq/m^3^ during winter), therefore pointing to a significant hazard posed both by the construction material used and by the volcanic substrate underneath it (the house actually stands on a thick layer of volcanic rocks).

Finally, as regards the two schools monitored, indoor radon concentration measured both in the short- and long-term were substantially reassuring. In the school at Giarre, in particular, where measurements were carried out in two different classes during the winter-spring months, radon concentration was consistently lower than 90 Bq/m^3^ in the short-term and lower than 55 Bq/m^3^ in the long-term. In the school at Zafferana Etnea, however, the data collected so far refer only to just over a month and are therefore not very representative, even if they show long-term radon concentration of only 31 Bq/m^3^.

A limitation of our study is the not large number of homes monitored; it is clear that more robust results can be obtained by extending the observations to a larger sample of dwellings. However, since it was the first time that indoor radon measurements were conducted over long periods in an active volcanic area such as Mt. Etna, we decided to focus our attention on long-term variations of indoor radon concentration in a few selected sites, in order to study what parameters possibly influence radon gas. This is very important, since it is known that radon emitted from the soil and infiltrated into houses varies mainly due to endogenous reasons, particularly in very active volcano-tectonic area such as Mt. Etna. Therefore, the results presented here unquestionably offer preliminary but interesting data for the evaluation of potentially severe health problems linked to the accumulation of radon in inhabited places, raising an issue so far unknown at Etna.

## Conclusions

Our study represents the first multi-year continuous indoor radon monitoring in houses located on the slopes of Mt. Etna volcano, Italy, where about one million people live. The aim was to verify if the houses built on the flanks of the volcano are prone to notable accumulation of radon inside them, as this is a dangerous carcinogenic gas.

Our measurements were performed during periods ranging from a few months to over 3 years in 12 rooms belonging to seven different houses distributed on the eastern, southern and south-western flanks of the volcano, located at different distances from seismogenic faults. In the long term (> 1 year), seven sensors showed radon concentration > 100 Bq/m^3^ and two showed radon concentration higher than 300 Bq/m^3^. In one case, indoor radon concentration reached an impressive value of 3,549 Bq/m^3^ and it was > 1,000 Bq/m^3^ for several consecutively months.

The most relevant result is that the highest concentration of indoor radon was recorded in locations closer to active faults (40–150 m) and above volcanic substrates (succession of lava flows > 60 m thick).

**Figure 11:**
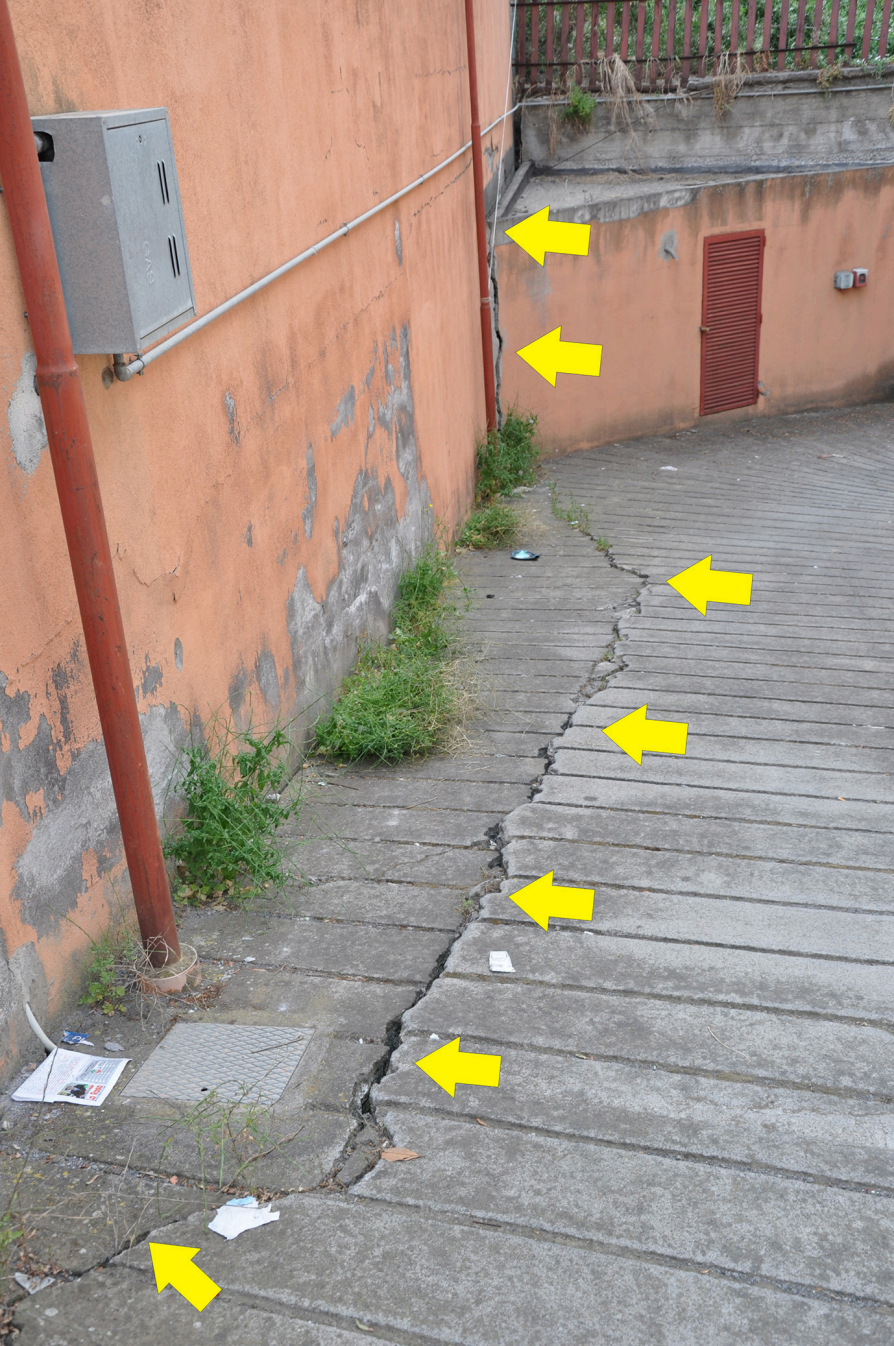
Example of active faulting (indicated by yellow arrows) affecting the urban area of Aci Trezza (see ATF in Figure 1 for location). These fractures constitute a preferential path for the ascent of crustal and sub-crustal gases, including radon, toward the surface and hence into houses.

Building materials (load-bearing walls in lava stone) were found to have some kind of effect on radon accumulation. This increases the geo-hazard of the studied areas, because they are not only affected by active faulting (Figure 11), seismicity and the related soil gas emissions, but they are also affected by high radon emissions that can infiltrate the houses.

Our temporal monitoring showed that radon concentration was generally lower in the summer, when air temperature is higher. In the two cases where this atmospheric effect was not found, we can hypothesize a possible endogenous cause, i.e., volcanic and/or tectonic activity of Mt. Etna. This result is quite surprising, as it opens up a new and never previously hypothesized field of investigation, in which even indoor measurements, under certain conditions, can provide useful data for monitoring endogenous phenomena. Another important finding was that the distance of monitoring sites from faults, and hence the intensity of radon emission from soil, is apparently a major parameter controlling the accumulation of radon indoor even during periods of favorable weather conditions (i.e., with high air temperature and low air density), thus overwhelming any possible mitigation from unforced air convection into houses.

Finally, our daily measurements showed high accumulations of indoor radon when the houses remained closed for a few days; this stresses the importance of frequently ventilating homes as an effective prevention action, especially in areas characterized by intense diffuse degassing of radon from the ground. Our results demonstrate the need to continue these studies, extending long-term indoor radon measurements to other urban areas, in order to assess the radon hazard for the ever-growing population living on Mt. Etna.

## Author Contributions

MN coordinated the research and largely wrote the manuscript. MN and AL collected and synthesized data. SG performed statistical data analysis. All authors contributed ideas and input to the research and writing of the paper.

### Conflict of Interest Statement

The authors declare that the research was conducted in the absence of any commercial or financial relationships that could be construed as a potential conflict of interest.
